# Future Implications of Artificial Intelligence in Medical Education

**DOI:** 10.7759/cureus.51859

**Published:** 2024-01-08

**Authors:** Forrest Bohler, Nikhil Aggarwal, Garrett Peters, Varna Taranikanti

**Affiliations:** 1 Foundational Medical Studies, Oakland University William Beaumont School of Medicine, Rochester, USA

**Keywords:** medical school education, medical education technology, medical resident education, medical education curriculum, artificial intelligence in radiology, artificial intelligence and education, artificial intelligence in medicine

## Abstract

Artificial intelligence has experienced explosive growth in the past year that will have implications in all aspects of our lives, including medicine. In order to train a physician workforce that understands these new advancements, medical educators must take steps now to ensure that physicians are adequately trained in medical school, residency, and fellowship programs to become proficient in the usage of artificial intelligence in medical practice. This manuscript discusses the various considerations that leadership within medical training programs should be mindful of when deciding how to best integrate artificial intelligence into their curricula.

## Editorial

Experts tell us that artificial intelligence (AI) will affect nearly every facet of life within the coming decades, medicine and education included [1]. AI will revolutionize the practice of medicine, but its effects are likely to be unequally felt across specialties [2,3]. For some fields of medicine, AI is likely to have a positive impact on a physician's ability to practice medicine through its ability to enhance physician efficiency and productivity. For example, gastroenterologists spend years of fellowship training honing their ability to accurately detect and resect cancer lesions lining colon mucosa through endoscopic procedures. This visual recognition is prone to human error, however, resulting in roughly 25% of all pre-cancerous lesions going undetected by gastroenterologists [4]. AI has been trained to recognize these lesions with greater specificity than a gastroenterologist alone [2]. In the coming years, the use of this technology may become the standard of care for colorectal cancer screenings. Although AI will greatly enhance the quality of care delivered, it is unlikely that AI will be able to fully replace gastroenterologists due to the many other procedural skills involved in colorectal cancer screenings that cannot be replicated by AI.

For other specialties where the bulk of care is focused on visual and pattern recognition, such as pathology and diagnostic radiology, AI will be more disruptive. Already, AI has the ability to screen and diagnose a computerized tomography scan in a fraction of the time it would take a radiologist [3]. While there will always be a need for individuals trained in pathology and diagnostic radiology, AI will likely drastically reduce the workload for these physicians beyond what the market can handle to support the current workforce of these physicians. 

The roles educators will play in teaching of AI applications will be many. One of the initial topics educators must make students aware of is the prognosis of various medical specialties in a future that will be dominated by AI. Students must be taught the issues that will be faced by specialists in fields such as pathology so that they do not fall victim to professional obsolesce. At the very least, they must know the shifting dynamics within these fields so they can make an educated decision about which specialty to pursue after medical school. This will likely have a profound impact on graduate medical education as certain residency programs may struggle to fill their residency positions due to declining interest in select fields of medicine.

A more prominent role educators will play regarding AI is in its instructional use in the delivery of medical care. Universities and residency programs that completely neglect the teachings of AI will not only be doing a disservice to its students and patients but also to themselves. Trainees entering a medical workforce with minimal knowledge of the use of relevant AI in clinical practice are likely to deliver inferior healthcare to their patients relative to their AI-savvy colleagues. Eventually, physicians trained without the proper application of AI technologies may find themselves delivering healthcare that is below the standard of care and could even risk legal issues involving malpractice and, eventually, forfeiture of their medical license. Graduate medical education programs that do not quickly integrate AI into their training programs are also likely to fall behind other more forward-thinking programs and subsequently fail to attract residents and fellows to train at their institutions. The importance of the incorporation of this new technology is underscored via survey findings that reveal the majority of medical students believe medical curricula must be updated in ways that effectively train future physicians on how to best utilize AI in their medical practice [5].

While some institutions may consciously avoid integrating AI into their training programs, others with fewer resources may struggle to afford the increased costs associated with this new technology, calling into question the issue of AI and equity in medical education. Many under-resourced training programs are located in areas that serve marginalized communities such as rural and minority populations, and the trainees educated at these programs are more likely to practice and serve these populations as attending physicians. If these physicians exit residency with inferior levels of training, exacerbations in health discrepancies among already marginalized groups are likely to be observed. This should be addressed by our nation's leaders through concerted efforts to fund these programs and hedge the potential for these increased inequities in healthcare. Within state-funded public medical schools, state legislators should consider an increased allocation of their state's budget towards these institutions to account for the increased costs associated with AI technology. Funding for AI within private institutions may prove more difficult and costly for students as the bulk of private medical schools' operation costs are derived from student tuition and fees. Subsequent increases in tuition for these schools would likely create further barriers in access to medical education, especially for disadvantaged students coming from lower socioeconomic backgrounds. For graduate medical education, however, increased funding for residency and fellowship programs should be addressed at the federal level, as the majority of funding for these programs is derived from Medicare. 

While the use of AI in training programs is imperative, educators must strike a balance in how they integrate its use into their teachings to avoid an overreliance on this new technology at the expense of innate clinical skills. AI has made great strides in clinical practice, yet it is not without its shortcomings and biases. The knowledge of a physician is still needed to address these limitations, but this knowledge is unlikely to be gained if the totality of a trainee's education contains elements of incorporated AI. For example, a gastroenterology fellow trained exclusively on AI technology that identifies pre-cancerous lesions in colorectal screenings is unlikely to develop the ability to identify polyps that were missed by AI. Additionally, as AI systems depend on large datasets to diagnose and predict disease conditions, any novel or rare disease may be difficult to interpret with limited data available on AI systems, underscoring the continued need for physicians' expertise in the diagnosis. For these reasons, educators in leadership positions must be keenly aware of this paradigm so that they can design curricula that optimize clinical skill development with proficiency in AI technology.

AI has the potential to revolutionize medicine, but only if we are deliberate in how we incorporate this new technology into the training of future physicians. We must start with medical educators as they will become the pacesetters in the years to come through their development of the future physician workforce. 
